# Severity of Osteoarthritis Is Associated with Increased Arterial Stiffness

**DOI:** 10.1155/2016/6402963

**Published:** 2016-07-17

**Authors:** Kaspar Tootsi, Jaak Kals, Mihkel Zilmer, Kaido Paapstel, Aare Märtson

**Affiliations:** ^1^Department of Traumatology and Orthopaedics, University of Tartu, Puusepa Street 8, 51014 Tartu, Estonia; ^2^Endothelial Centre, University of Tartu, Tartu, Estonia; ^3^Department of Surgery, University of Tartu, Tartu, Estonia; ^4^Institute of Biomedicine and Translational Medicine and Department of Biochemistry, Centre of Excellence for Genomics and Translational Medicine, University of Tartu, Ravila Street 19, 50411 Tartu, Estonia; ^5^Traumatology and Orthopaedics Clinic, Tartu University Hospital, Tartu, Estonia

## Abstract

*Objective.* Osteoarthritis (OA) is associated with increased cardiovascular comorbidity and mortality. Evidence is lacking about whether arterial stiffness is involved in OA. The objective of our study was to find out associations between OA, arterial stiffness, and adipokines.* Design.* Seventy end-stage knee and hip OA patients (age 62 ± 7 years) and 70 asymptomatic controls (age 60 ± 7 years) were investigated using the applanation tonometry to determine their parameters of arterial stiffness. Serum adiponectin, leptin, and matrix metalloproteinase 3 (MMP-3) levels were determined using the ELISA method. Correlation between variables was determined using Spearman's rho. Multiple regression analysis with a stepwise selection procedure was employed.* Results.* Radiographic OA grade was positively associated with increased carotid-femoral pulse wave velocity (cf-PWV) (*r* = 0.272, *p* = 0.023). We found that OA grade was also associated with leptin and MMP-3 levels (rho = −0.246, *p* = 0.040 and rho = 0.235, *p* = 0.050, resp.). In addition, serum adiponectin level was positively associated with augmentation index and inversely with large artery elasticity index (rho = 0.293, *p* = 0.006 and rho = −0.249, *p* = 0.003, resp.).* Conclusions.* Our results suggest that OA severity is independently associated with increased arterial stiffness and is correlated with expression of adipokines. Thus, increased arterial stiffness and adipokines might play an important role in elevated cardiovascular risk in end-stage OA.

## 1. Introduction

Osteoarthritis (OA) is the most common form of arthritis and a growing burden on the public healthcare [1]. Evidence has emerged that patients with OA have increased cardiovascular (CV) morbidity and mortality [1, 2]; however, very little is known about the exact mechanism underlying this association. Several potential mechanisms such as obesity, chronic systemic inflammation, balance of adipokine levels, limited physical activity, and use of nonsteroid anti-inflammatory drugs might be involved in the elevated risk of CV disease (CVD) in OA [3, 4].

The adipokines are white-adipose-tissue derived cell signaling proteins that participate in multiple homeostasis maintaining functions [5]. Evidence is accumulating on multiple adipokines among which leptin and adiponectin have been most thoroughly investigated. Leptin has been associated with CV events, mortality, and diabetes [6, 7]. Adiponectin has been shown to have anti-inflammatory, antiatherogenic, and cardiomyocyte protective properties in experimental settings [8]. However, in human population this role seems to be far more complex as higher concentrations have been linked to increased CV morbidity and mortality [9]. In addition, leptin and adiponectin are also involved in the pathogenesis of OA. Studies have shown that OA chondrocytes produce more leptin inducing thereby the production of cartilage degradation enzymes [10]. Adiponectin seems to possess a protective role in OA through downregulating inflammatory mediators and upregulating protease inhibitors [11].

Arterial stiffness has been shown to be an independent determinant of CV morbidity and mortality and has been proposed as a surrogate endpoint for CVD [12]. Arterial stiffness can be measured noninvasively and cf-PWV as a marker of aortic stiffness is considered the gold standard method [12]. It is noteworthy that systemic inflammation and adipokines that have been associated with OA are also involved in the pathogenesis of arterial stiffness. Aortic elastic properties have been found to be elevated in radiographically established osteoarthritis [13]. At the same time, no association has been found between knee bone marrow lesions and cf-PWV [14]. Evidence on arterial stiffness and osteoarthritis is so far lacking or inconclusive.

A better understanding of the associations between OA, adipokines, and CVD might help extend future management strategies of OA beyond the current focus on treating only chronic symptoms prior to total joint replacement. The aim of this study was to find out whether OA is associated with arterial stiffness and adipokine levels.

## 2. Methods

### 2.1. Study Population

This cross-sectional study included 70 patients with primary end-stage hip and knee OA, who presented for total joint arthroplasty, and 70 age and gender matched controls. The OA patients were elected from the Traumatology and Orthopaedics Clinic of Tartu University Hospital prior to hip and knee replacement surgery. All patients were diagnosed according to the American College of Rheumatology criteria for knee and hip OA [15, 16]. Our study had focused predominantly on the systemic effect of OA. Patients with any acute or chronic inflammatory disease, diabetes, coronary artery disease, cardiac arrhythmias or known valve pathology, peripheral atherosclerotic disease, malignancies, or renal insufficiency (eGFR < 60 mL/min/1.73 m^2^) were excluded.

Age and gender matched apparently healthy controls were recruited through local family physicians in the same geographical region. The exclusion criteria for the control group were the following: any acute or chronic inflammatory disease, any persistent knee or hip joint pain, a visit to family practitioner because of hip or knee joint complaints, diabetes, hypertension, coronary artery disease, cardiac arrhythmia, cerebrovascular or peripheral artery disease, malignancies, renal insufficiency, and regular use of any medication.

The study was conducted in accordance with the Helsinki declaration and was approved by the Ethics Committee of the University of Tartu. Written informed consent was obtained from all participants.

### 2.2. Study Protocol

Blood samples were collected between 07:00 and 11:00 after an overnight fast and abstinence from tobacco and alcohol. The height and weight and waist and hip circumference of the patients were recorded and joint-specific evaluation was performed. The Harris Hip Score (HHS) [17] was used for hip joint evaluation and the Hospital for Special Surgery (HSS) Knee Score [18] was used for knee joint evaluation. Both of these test results range from 0 to 100, where 100 is the best possible outcome. After 10–15 minutes of rest in a temperature controlled quiet room in a supine position, the participants' blood pressure and pulse wave velocity were recorded and pulse wave analysis was made. All pulse wave parameters were measured at least in duplicate and the average was calculated.

### 2.3. Arterial Stiffness and Central Hemodynamic Measurements

Pulse wave analysis and velocity were used to measure arterial stiffness. Left radial artery waveforms were recorded with a high fidelity micromanometer (SPT-301BH, Millar Instruments, TX, USA). Corresponding ascending aortic waveforms were then generated using a transfer function to calculate central hemodynamics and augmentation index (AIx). The cf-PWV and carotid-radial pulse wave velocity (cr-PWV) were measured by sequentially recording ECG-gated carotid and femoral or radial artery waveforms using a SphygmoCor device (SphygmoCor, AtCor Medical, Sydney, Australia). The pulse waveform was recorded from the right radial artery with a Cardiovascular Profiling Instrument (HDI/PulseWave CR-2000, Hypertension Diagnostics Inc., Eagan, USA) and large (C1) and small (C2) artery elasticity indices were recorded.

### 2.4. Biochemical Analysis

Venous blood samples were centrifuged at room temperature and the serum was stored at −70°C until analysis. Serum adiponectin levels were measured using an enzyme-linked immunosorbent assay (ELISA) (Human Total Adiponectin/Acrp30 Immunoassay, R&D Systems Europe, Abingdon, UK). Human Total MMP-3 Immunoassay kits available from R&D systems a Bio-Techne brand (catalogue number DMP 300) were used for quantitative determination of human active and promatrix metalloproteinase 3 (total pro-MMP-3) concentrations. The level of serum leptin was determined using The Evidence Investigator (Metabolic Syndrome Array-1, Randox Laboratories, Crumlin, UK). The serum levels of triglycerides, total cholesterol, LDL-cholesterol, HDL-cholesterol, white blood cell (WBC) count, high-sensitivity C-reactive protein (hs-CRP), serum creatinine, and eGFR were measured using standard laboratory methods in the local clinical laboratory with automated analyzers.

### 2.5. Radiographic Assessment

Standard weight-bearing anteroposterior knee and hip joint radiographs were taken. Osteoarthritis was assessed according to the Kellgren-Lawrence grading system, where 0 represents no changes; 1 represents doubtful joint space narrowing; 2 represents definite osteophytes and doubtful joint space narrowing; 3 represents definite osteophytes, joint space narrowing, sclerosis, and possible deformity; and 4 represents marked joint space narrowing, large osteophytes, severe sclerosis, and definite bone deformity [19]. The radiographs were evaluated independently by two raters blinded to the clinical data and a consensus score was produced. Intraclass correlation coefficient (ICC) was used to assess the consistency among the raters. The ICC was found to be 0.76, 95% CI (0.61–0.85), which is considered good.

### 2.6. Statistical Analysis

Statistical analysis of data was performed by SPSS 22.0 (SPSS Inc., Chicago IL, USA) for Windows. The Mann-Whitney* U* test was used for comparing means between two groups. Continuous variables are presented as mean ± SD. Since there was a significant difference of BMI and blood pressure between the study groups, the levels of PWV were adjusted for BMI and mean arterial pressure and the levels of adipokines were adjusted for BMI before analysis. Spearman's rho was used for determining correlation between variables. A chi-square test or Fischer's exact test was used to compare group proportions where appropriate. Stepwise multiple regression analysis was performed to determine independent associations between variables.

## 3. Results

A total of 140 subjects (women 49%, aged 49–79, and mean 61 years) participated in the study. General characteristics of the two study groups are presented in Table 1. The level of BMI and the W/H-ratio were significantly higher, and HHS and HSS Knee Scores were lower for the OA group. Smoking status did not differ significantly between the study groups. Hemodynamic parameters are presented in Table 2. Peripheral and central blood pressure were higher in the OA group. Since there was a discrepancy in BMI and blood pressure (a determinant of PWV) between the study groups, the corresponding hemodynamic and biochemical variables were adjusted for mean arterial pressure and BMI. The cf-PWV was higher and C2 was lower for the OA group compared to the controls. The levels of leptin, WBC, hs-CRP, and urea were higher and adiponectin and HDL-cholesterol were lower for the patient group (Table 3). However, after correcting for BMI, the difference in hs-CRP and platelet count was no longer significant and of borderline significance for adiponectin.

**Figure 1:**
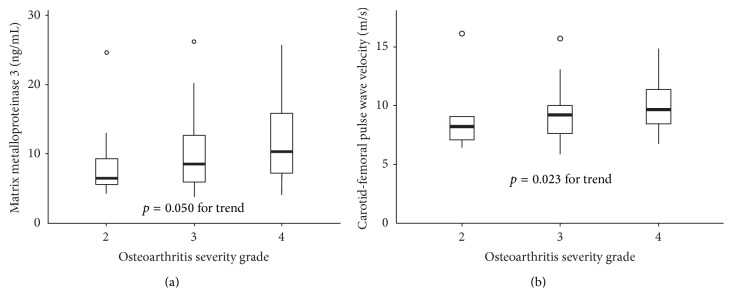
(a) Boxplot describing cf-PWV in different groups of osteoarthritis severity. (b) Matrix metalloproteinase 3 levels for different osteoarthritis severity scores.

A significant positive association was found between OA radiographic Kellgren-Lawrence grade and cf-PWV (*r* = 0.272, *p* = 0.023) (Figure 1(a)). The association was further analyzed separately in the hip and knee subgroups (*r* = 0.396, *p* = 0.011 and *r* = 0.351, *p* = 0.062, resp.). Multiple linear regression analysis using a stepwise selection procedure was performed to study the association between OA grade and cf-PWV. In the hip OA group cf-PWV was set as the dependent variable, and OA grade, age, mean arterial pressure (MAP), and hs-CRP remained significant independent predictors (Table 4(a)). In the knee OA group, after adjusting for W/H-ratio and age, OA grade was not an independent predictor of cf-PWV (Table 4(b)). In addition, Kellgren-Lawrence grade correlated positively with MMP-3 (*r* = 0.235, *p* = 0.05) (Figure 1(b)) and negatively with leptin (*r* = −0.246, *p* = 0.040). However, these associations were not significant after adjustment for potential confounders.

**Figure 2:**
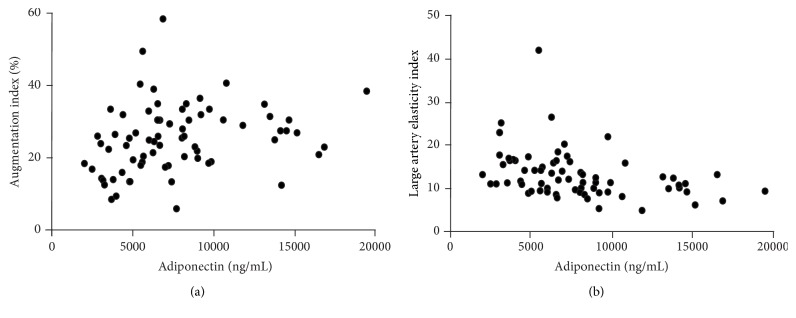
Associations of adiponectin with (a) augmentation index (rho = 0.293, *p* = 0.006) and (b) large artery elasticity index (rho = −0.249, *p* = 0.003).

Furthermore, we found that adiponectin was correlated with C1 and AIx@75 (*r* = −0.249, *p* = 0.003 and *r* = 0.293, *p* < 0.001, resp.) (Figure 2). In the model where adiponectin was set as the dependent variable, neither C1 nor AIx were independent predictors when potential confounders were included (data not shown).

## 4. Discussion

Our study describes associations between adipokines, MMP-3, and parameters of arterial stiffness in older adults with hip and knee OA and compares them with the corresponding indicators for asymptomatic controls. To our knowledge, this study is the first to demonstrate independent correlation between radiographic OA grade and cf-PWV. These findings support the notion that arterial stiffening might be linked to the pathogenesis of OA. In addition, we demonstrate that adipokines are associated with OA and with arterial stiffness and might thus play a role in linking these two pathologies.

Emerging evidence suggests that stiffening of the arteries is related to the OA. Recently, a study of 80 end-stage knee OA patients found decreased elastic properties of aorta compared to non-OA controls [13]. In addition, the authors found that radiographic severity of OA was associated with aortic stiffness. However, another study of knee OA patients found no relationship between presence or size of bone marrow lesions (a surrogate for OA) and cf-PWV [14]. Hand OA has been linked to arterial stiffness, but the relationship was largely attributable to the confounding effect of age [3]. So far the relevant evidence has been controversial and to the best of our knowledge data concerning hip OA and arterial stiffness is lacking. In the present study of knee and hip OA we demonstrated independent association between OA severity and cf-PWV (Figure 1, Table 4). This finding supports the association between OA severity and arterial stiffness. We studied patients with hip OA, which is considered a different entity of OA [20], and found its independent association with cf-PWV. The reason for failing to reveal significant association between knee OA severity and cf-PWV might be attributed to too small sample size. Yet we established relationship between hip OA and arterial stiffness, which might contribute to the higher CVD risk among OA population.

The adipokines are a growing family of white-adipose-tissue derived factors that have multiple functions through different pathways and are involved in inflammation and modulation of immunological response but also in glucose and lipid metabolism [4]. Many adipokines have been associated with OA. Leptin has proinflammatory properties and complex actions on chondrocytes. Leptin upregulates MMPs and induces cartilage loss [21–23]; however, its anabolic effects have also been described [24]. Although leptin has been associated with OA severity [25], the pathophysiological pathways are not fully understood. In addition to bone and cartilage, leptin influences also the cardiovascular system. It is an independent predictor of myocardial infarction and stroke and has been associated with increased arterial stiffness [26] through promoting inflammation and MMP upregulation [27]. In accord with previous studies, we found an elevated expression of leptin in OA patients and, in addition, an inverse association with OA radiographic grade, which might indicate the anabolic effect of leptin. These findings point to the possible key role of leptin in the pathogenesis of OA and suggest leptin as a link between OA and vascular damage.

Adiponectin is prevalently synthesised in adipose tissue and circulates in the blood in large quantities [28]. Adiponectin has anti-inflammatory properties and hypoadiponectinemia has a detrimental effect on aortic stiffness [29]. In the present study, adiponectin correlated inversely with large artery elasticity index and positively with AIx@75. To the best of our knowledge, adiponectin's interactions with arterial stiffness have not been investigated before in OA patients. Findings similar to ours were reported in essential hypertension [30], but inverse association was recently found in juvenile idiopathic arthritis patients [31]. Our results suggest that adiponectin as well as leptin might link OA with increased arterial stiffness. These findings describe the pleiotropic role of adipokines in OA and need further research to determine their potential as therapeutic targets.

The MMP-3 is a catabolic enzyme that not only has the ability to degrade the extracellular matrix but plays a central role in activating other members of the MMP family. We found higher level of MMP-3 in OA patients compared to controls and positive correlation between radiographic grade and serum level of MMP-3 in OA subjects. The activation and secretion of MMP-3 are driven by inflammatory cytokines, which in turn enhances the production of inflammatory mediators like interleukin-1 [32]. This is in line with our results, according to which OA group also showed higher values of WBC, which indicates a systemic inflammatory state in these patients. Furthermore, MMP-3 is likely to influence arterial stiffness and has been found to be elevated in atherosclerotic aorta [33, 34]. In conclusion, MMP-3 might be another marker that is associated with OA and vascular pathology.

Our study has some limitations that should be recognized. First, because of the cross-sectional study design, causal associations could not be established. Second, since X-rays for the control group were not available, some asymptomatic OA cases might be included in the controls. Third, data concerning physical activity was not collected but has been shown to influence arterial stiffness and, therefore, is a confounding factor that was not accounted for in the present study.

In conclusion, our study demonstrates independent association between OA radiographic severity and aortic stiffness. We also found that adipokines are related to OA severity. These relationships provide a potential link between biochemical and functional alterations and OA as well as elevated CV risk in end-stage OA.

## Figures and Tables

**Table 1:** General parameters of the osteoarthritis patients and the controls.

Variable	Osteoarthritis (*n* = 70)	Controls (*n* = 70)	*p* value
Age (years)	62 ± 7	60 ± 7	0.066
Male/female (*n*)	35/35	36/34	0.866
BMI (kg/m^2^)	28 ± 3	26 ± 3	0.001
Waist circumference (cm)	96 ± 9	88 ± 11	<0.001
Hip circumference (cm)	104 ± 7	102 ± 6	0.033
W/H-ratio	0.92 ± 0.08	0.87 ± 0.09	0.001
Smoking (*n*, %)			0.127
Current smoker	15 (22)	8 (11)	
Former smoker	4 (6)	9 (13)	
Nonsmoker	50 (72)	53 (76)	
Involved joint (*n*)			
Hip	41	0	
Knee	29	0	
Harris Hip Score	37 (32–48)	100 (100–100)	<0.001
HSS Knee Score	62 (49–65)	100 (100–100)	<0.001

BMI: body mass index, W/H-ratio: waist-to-hip ratio, and HSS: Hospital for Special Surgery.

The values are expressed as means ± SD or medians (with interquartile range).

**Table 2:** Hemodynamic parameters of the osteoarthritis patients and the controls.

Variable	Osteoarthritis patients (*n* = 70)	Controls (*n* = 70)	*p* value
Peripheral SBP (mm Hg)	133 ± 18	126 ± 15	0.011
Peripheral DBP (mm Hg)	81 ± 8	78 ± 7	0.014
Central SBP (mm Hg)	125 ± 17	118 ± 15	0.014
Central DBP (mm Hg)	82 ± 8	78 ± 8	0.019
Mean pressure (mm Hg)	100 ± 11	95 ± 10	0.010
Heart rate (bpm)	64 ± 11	61 ± 7	0.042
AIx@75 (%)	25 ± 9	22 ± 11	0.078
cf-PWV (m/s)	9.1 ± 2.2	8.2 ± 1.5	0.007^a^
C1 (mL/mm Hg × 100)	12.8 ± 4.4	14.1 ± 5.0	0.119
C2 (mL/mm Hg × 100)	4.2 ± 3.6	6.0 ± 2.7	0.002

SBP: systolic blood pressure, DBP: diastolic blood pressure, AIx@75: augmentation index corrected for a heart rate of 75 beats per minute, cf-PWV: carotid to femoral pulse wave velocity, C1: large artery elasticity index, and C2: small artery elasticity index.

All variables are adjusted for body mass index.

^a^Adjusted for mean arterial pressure.

**Table 3:** Metabolic markers for the osteoarthritis and control groups.

Variable	Osteoarthritis patients (*n* = 70)	Controls (*n* = 70)	*p* value
Triglycerides (mmol/L)	1.6 ± 1.2	1.4 ± 0.8	0.249
LDL-cholesterol (mmol/L)	4.0 ± 0.9	3.9 ± 1.0	0.630
HDL-cholesterol (mmol/L)	1.5 ± 0.4	1.7 ± 0.5	0.026
Total cholesterol (mmol/L)	5.8 ± 1.1	5.8 ± 1.2	0.892
hs-CRP (mg/L)	1.89 ± 1.14	1.42 ± 0.96	0.093
eGFR (mL/mg/1.73 m^2^)	83 ± 15	83 ± 12	0.991
Urea (mmol/L)	6.0 ± 2.0	5.2 ± 1.2	0.017
White blood cells (10^9^/L)	6.5 ± 1.4	5.7 ± 1.9	0.010
Platelets (10^9^/L)	243 ± 57	227 ± 55	0.123
MMP-3 (ng/mL)	14.1 ± 6.54	10.9 ± 5.94	0.004
Adiponectin (ng/mL)	8131 ± 5681	9664 ± 3897	0.066
Leptin (ng/mL)	3.7 ± 5.14	2.20 ± 1.85	0.022

LDL: low-density lipoprotein, HDL: high-density lipoprotein, hs-CRP: high-sensitivity C-reactive protein, eGFR: estimated glomerular filtration rate, and MMP-3: matrix metalloproteinase 3.

All variables are adjusted for body mass index.

**(a):** 

Variable	*B*	Std error	Beta	*t*	*p* value
*Constant*	−8.487	2.445		−3.471	0.001
OA grade	0.739	0.332	0.219	2.228	0.033
Age	0.125	0.029	0.422	4.302	<0.001
MAP	0.079	0.016	0.477	4.880	<0.001
hs-CRP	−0.433	0.170	−0.246	−2.543	0.016

**(b):** 

Variable	*B*	Std error	Beta	*t*	*p* value
*Constant*	−10.450	5.357		−1.951	0.062
Age	0.164	0.047	0.556	3.492	0.002
W/H-ratio	11.326	5.345	0.344	2.119	0.044
OA grade	−0.227	0.695	−0.055	−0.327	0.746

OA: osteoarthritis, MAP: mean arterial pressure, hs-CRP: high-sensitivity C-reactive protein, and W/H-ratio: waist-to-hip ratio.

## References

[1] Rahman M. M., Kopec J. A., Anis A. H., Cibere J., Goldsmith C. H. (2013). Risk of cardiovascular disease in patients with osteoarthritis: A Prospective Longitudinal Study. *Arthritis Care and Research*.

[2] Nüesch E., Dieppe P., Reichenbach S., Williams S., Iff S., Jüni P. (2011). All cause and disease specific mortality in patients with knee or hip osteoarthritis: Population based cohort study. *British Medical Journal*.

[3] Saleh A. S., Najjar S. S., Muller D. C. (2007). Arterial stiffness and hand osteoarthritis: a novel relationship?. *Osteoarthritis and Cartilage*.

[4] Gualillo O., González-Juanatey J. R., Lago F. (2007). The emerging role of adipokines as mediators of cardiovascular function: physiologic and clinical perspectives. *Trends in Cardiovascular Medicine*.

[5] Bouziana S., Tziomalos K., Goulas A., Hatzitolios A. I. (2016). The role of adipokines in ischemic stroke risk stratification. *International Journal of Stroke*.

[6] Ku I. A., Farzaneh-Far R., Vittinghoff E., Zhang M. H., Na B., Whooley M. A. (2011). Association of low leptin with cardiovascular events and mortality in patients with stable coronary artery disease: The Heart and Soul Study. *Atherosclerosis*.

[7] Welsh P., Murray H. M., Buckley B. M. (2009). Leptin predicts diabetes but not cardiovascular disease: results from a large prospective study in an elderly population. *Diabetes Care*.

[8] Turer A. T., Scherer P. E. (2012). Adiponectin: mechanistic insights and clinical implications. *Diabetologia*.

[9] Persson J., Folkersen L., Ekstrand J. (2012). High plasma adiponectin concentration is associated with all-cause mortality in patients with carotid atherosclerosis. *Atherosclerosis*.

[10] Poonpet T., Honsawek S. (2014). Adipokines: biomarkers for osteoarthritis?. *World Journal of Orthopaedics*.

[11] Chen T.-H., Chen L., Hsieh M.-S., Chang C.-P., Chou D.-T., Tsai S.-H. (2006). Evidence for a protective role for adiponectin in osteoarthritis. *Biochimica et Biophysica Acta—Molecular Basis of Disease*.

[12] Townsend R. R., Wilkinson I. B., Schiffrin E. L. (2015). Recommendations for improving and standardizing vascular research on arterial stiffness: a scientific statement from the American Heart Association. *Hypertension*.

[13] Belen E., Karaman O., Caliskan G., Atamaner O., Aslan O. (2016). Impaired aortic elastic properties in primary osteoarthritis. *Vascular*.

[14] Goldsmith G. M., Aitken D., Cicuttini F. M. (2014). Osteoarthritis bone marrow lesions at the knee and large artery characteristics. *Osteoarthritis and Cartilage*.

[15] Altman R., Alarcón G., Appelrouth D. (1991). The American college of rheumatology criteria for the classification and reporting of osteoarthritis of the hip. *Arthritis and Rheumatism*.

[16] Altman R., Asch E., Bloch D. (1986). Development of criteria for the classification and reporting of osteoarthritis: classification of osteoarthritis of the knee. *Arthritis & Rheumatism*.

[17] Harris W. H. (1969). Traumatic arthritis of the hip after dislocation and acetabular fractures: treatment by mold arthroplasty. An end-result study using a new method of result evaluation. *Journal of Bone and Joint Surgery—Series A*.

[18] Insall J. N., Ranawat C. S., Aglietti P., Shine J. (1976). A comparison of four models of total knee-replacement prostheses. *The Journal of Bone & Joint Surgery—American Volume*.

[19] Kellgren J. H., Lawrence J. S. (1957). Radiological assessment of osteo-arthrosis. *Annals of the Rheumatic Diseases*.

[20] Cushnaghan J., Dieppe P. (1991). Study of 500 patients with limb joint osteoarthritis. I. Analysis by age, sex, and distribution of symptomatic joint sites. *Annals of the Rheumatic Diseases*.

[21] Koskinen A., Vuolteenaho K., Nieminen R., Moilanen T., Moilanen E. (2011). Leptin enhances MMP-1, MMP-3 and MMP-13 production in human osteoarthritic cartilage and correlates with MMP-1 and MMP-3 in synovial fluid from oa patients. *Clinical and Experimental Rheumatology*.

[22] Dumond H., Presle N., Terlain B. (2003). Evidence for a key role of leptin in osteoarthritis. *Arthritis and Rheumatism*.

[23] Otero M., Gomez Reino J. J., Gualillo O. (2003). Synergistic induction of nitric oxide synthase type II: in vitro effect of leptin and interferon-*γ* in human chondrocytes and ATDC5 chondrogenic cells. *Arthritis and Rheumatism*.

[24] Berry P. A., Jones S. W., Cicuttini F. M., Wluka A. E., MacIewicz R. A. (2011). Temporal relationship between serum adipokines, biomarkers of bone and cartilage turnover, and cartilage volume loss in a population with clinical knee osteoarthritis. *Arthritis and Rheumatism*.

[25] Ku J. H., Lee C. K., Joo B. S. (2009). Correlation of synovial fluid leptin concentrations with the severity of osteoarthritis. *Clinical Rheumatology*.

[26] Gonzalez M., Lind L., Söderberg S. (2013). Leptin and endothelial function in the elderly: the Prospective Investigation of the Vasculature in Uppsala Seniors (PIVUS) study. *Atherosclerosis*.

[27] Scotece M., Conde J., Gómez R. (2012). Role of adipokines in atherosclerosis: interferences with cardiovascular complications in rheumatic diseases. *Mediators of Inflammation*.

[28] Kadowaki T., Yamauchi T. (2005). Adiponectin and adiponectin receptors. *Endocrine Reviews*.

[29] Tsioufis C., Dimitriadis K., Selima M. (2007). Low-grade inflammation and hypoadiponectinaemia have an additive detrimental effect on aortic stiffness in essential hypertensive patients. *European Heart Journal*.

[30] Mahmud A., Feely J. (2005). Adiponectin and arterial stiffness. *American Journal of Hypertension*.

[31] Ilisson J., Zagura M., Zilmer K. (2015). Increased carotid artery intima-media thickness and myeloperoxidase level in children with newly diagnosed juvenile idiopathic arthritis. *Arthritis Research and Therapy*.

[32] Chen J.-J., Huang J.-F., Du W.-X., Tong P.-J. (2014). Expression and significance of MMP3 in synovium of knee joint at different stage in osteoarthritis patients. *Asian Pacific Journal of Tropical Medicine*.

[33] Knox J. B., Sukhova G. K., Whittemore A. D., Libby P. (1997). Evidence for altered balance between matrix metalloproteinases and their inhibitors in human aortic diseases. *Circulation*.

[34] Agrotis A. (2005). The genetic basis for altered blood vessel function in disease: large artery stiffening. *Vascular Health and Risk Management*.

